# Supplementary material for: A novel approach to studying infective endocarditis: Ultrasound-guided wire injury and bacterial challenge in mice
Source: PLoS One. 2025 Apr 7;20(4):e0318955. doi: 10.1371/journal.pone.0318955 (PMC11975138; doi:10.1371/journal.pone.0318955)
Supplement: S1 Table — (DOCX) [file pone.0318955.s001.docx]

**A novel approach to studying Infective Endocarditis: Ultrasound-guided wire injury and bacterial challenge in mice**

Benedikt Bartsch*, Ansgar Ackerschott, Muntadher Al-Zaidi, Raul Nicolas Jamin, Mariam Louis Fathy Nazir, Moritz Altrogge, Lars Fester, Jessica Lambertz, Mark Coburn, Georg Nickenig, Sebastian Zimmer, Christina Katharina Weisheit


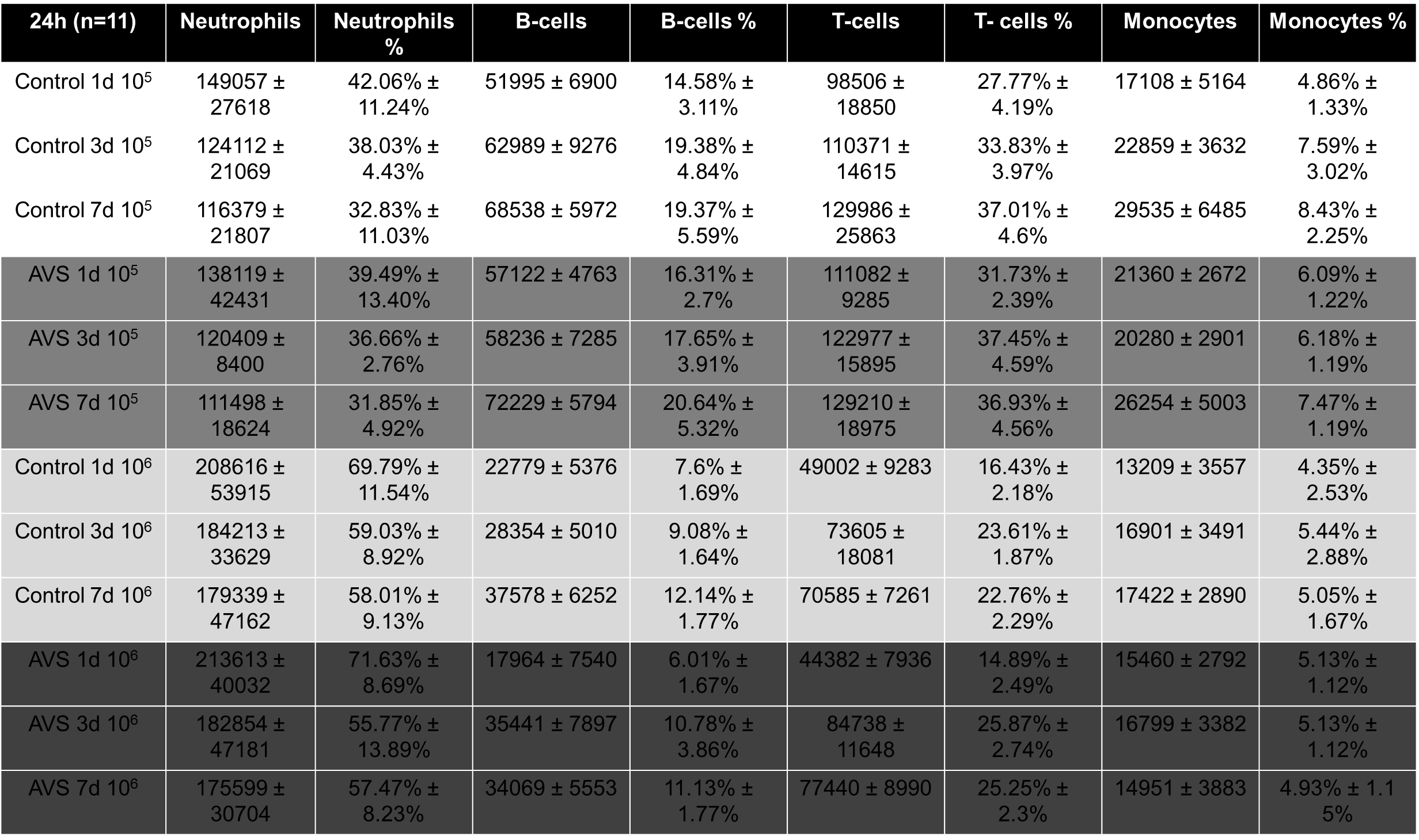


**Supplemental Table 1 Flow cytometry from murine blood samples**Blood samples were drawn before sacrifice either 1d, 3d or 7d after wire-injury. Bacterial challenge was performed 24h after wire-injury. Cells were stained for LD, B220, CD11b, CD45, Ly6G and CD3. Data is presented as mean ± SEM.
