# Supplementary material for: A novel approach to studying infective endocarditis: Ultrasound-guided wire injury and bacterial challenge in mice
Source: PLoS One. 2025 Apr 7;20(4):e0318955. doi: 10.1371/journal.pone.0318955 (PMC11975138; doi:10.1371/journal.pone.0318955)
Supplement: S1 Fig — (DOCX) [file pone.0318955.s002.docx]

**A novel approach to studying Infective Endocarditis: Ultrasound-guided wire injury and bacterial challenge in mice**

Benedikt Bartsch*, Ansgar Ackerschott, Muntadher Al-Zaidi, Raul Nicolas Jamin, Mariam Louis Fathy Nazir, Moritz Altrogge, Lars Fester, Jessica Lambertz, Mark Coburn, Georg Nickenig, Sebastian Zimmer, Christina Katharina Weisheit


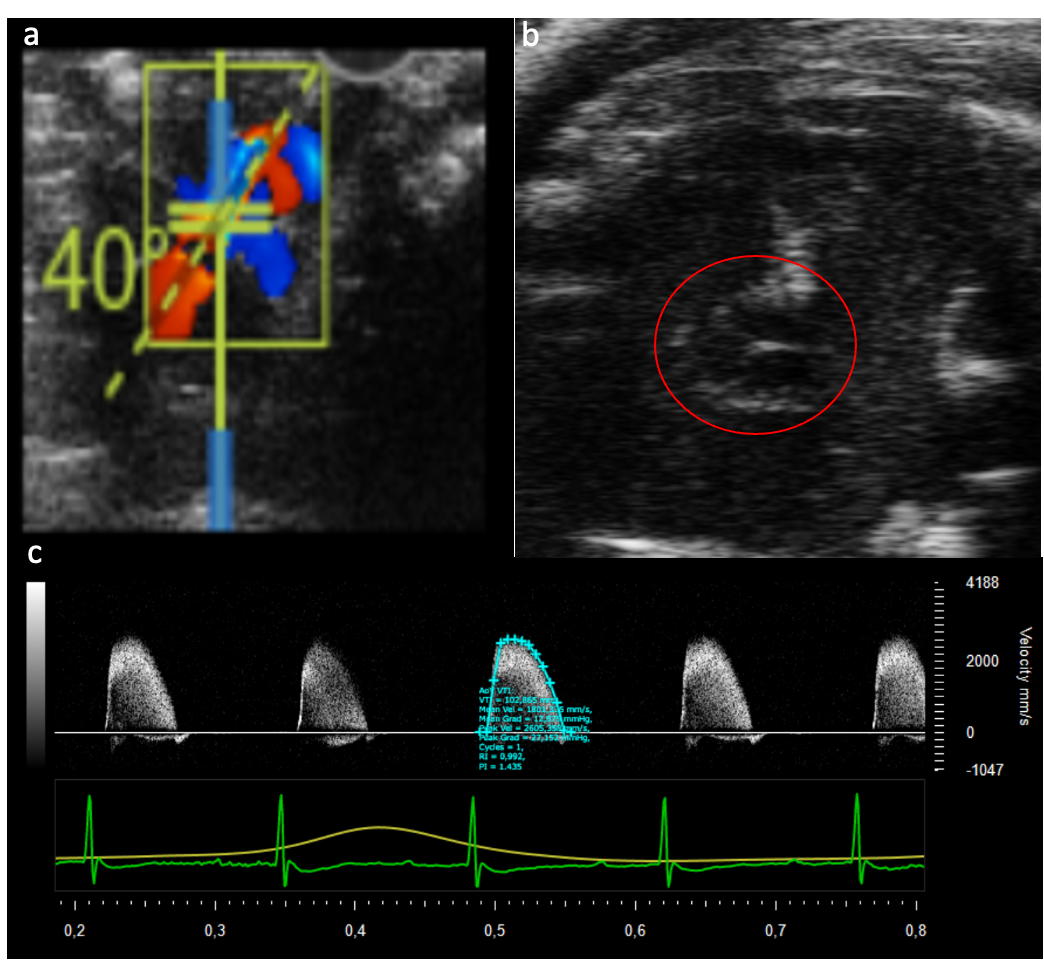

**Supplemental Figure 1 Exemplary images of echogradiographic analysis**
(a + c) Aortic valve peak velocity and gradients were measured in the suprasternal view with a pulse-wave-Doppler using angle correction between 40° and 50°. (b) Exemplary image of aortic valve cusp diameter in suprasternal short axis view.
